# A Three-Month Probiotic (the *Streptococcus salivarius* M18 Strain) Supplementation Decreases Gingival Bleeding and Plaque Accumulation: A Randomized Clinical Trial

**DOI:** 10.3390/dj12070222

**Published:** 2024-07-18

**Authors:** Ksenia Babina, Dilara Salikhova, Irina Makeeva, Alexandr Zaytsev, Inna Sokhova, Sevil Musaeva, Maria Polyakova, Nina Novozhilova

**Affiliations:** 1 Department of Therapeutic Dentistry, I.M. Sechenov First Moscow State Medical University (Sechenov University), Moscow 119991, Russia; salikhova_d_i@student.sechenov.ru (D.S.); makeeva_i_m@staff.sechenov.ru (I.M.); sokhova_i_a@staff.sechenov.ru (I.S.); svlmsv03@mail.ru (S.M.); polyakova_m_a_1@staff.sechenov.ru (M.P.); novozhilova_n_e@staff.sechenov.ru (N.N.); 2 Institute of Linguistics and Intercultural Communication, I.M. Sechenov First Moscow State Medical University (Sechenov University), Moscow 119991, Russia; zaytsev_a_b@staff.sechenov.ru

**Keywords:** dental plaque, gingivitis, probiotics, *Streptococcus salivarius* M18

## Abstract

*S. salivarius* M18 administration has been proven to provide positive effects on periodontal health; however, there is still no consensus on the optimum duration of probiotic administration. This study aimed to evaluate the effect of three months of probiotic supplementation on bleeding on probing, signs of gingival inflammation, and dental biofilm. Sixty-two eligible individuals with gingivitis were enrolled in this placebo-controlled, double-blind trial and randomly allocated to the M18 or control groups. Primary outcomes were changes in gingival condition (gingival index, GI; gingival bleeding index, GBI) after 1, 2, and 3 months of lozenges administration and after a one-month washout. Secondary outcomes included changes in the Quigley–Hein plaque index (modified by Turesky et al.) after 1, 2, and 3 months of lozenges administration and after a washout. In total, 60 individuals completed the study (31 and 29 in the M18 group and the control group, respectively). No severe adverse events were reported. Probiotic supplementation resulted in a significant decrease in gingival bleeding at 1 month (effect size 1.09 [CI95%: 0.55–1.63]), 2 months (effect size 0.78 [CI95%: 0.26–1.30]), and 3 months (effect size 0.67 [CI95%: 0.15–1.18]) and a significant reduction in dental plaque accumulation at 2 months (effect size 0.63 [CI95%: 0.12–1.14]) and 3 months (effect size 0.55 [CI95%: 0.03–1.05]). A three-month supplementation with the probiotic resulted in a significant reduction in gingival bleeding and biofilm accumulation; however, a long-lasting effect is not expected, indicating the need for probiotic intake on a long-term basis.

## 1. Introduction

Gingivitis is a reversible inflammatory process of the gingiva caused by dental plaque without noticeable bone loss or clinical attachment loss [1], which is characterized by a high prevalence worldwide [2,3]. At the onset, it manifests as minor alterations in the gingiva, and patients may note symptoms such as bleeding on brushing, gingival edema, and hyperemia; however, with the progression of the disease, the symptoms may become more pronounced, and patients may complain of tenderness and halitosis [2].

Dental plaque, representing a structured microbial biofilm, is a major etiologic factor causing gingivitis and dental caries. Therefore, it is of great importance to prevent its formation and accumulation on the surfaces of teeth and prostheses using effective plaque control measures [4].

Mechanical plaque removal is the primary and most effective measure to prevent periodontal disease [2,5,6]. Although educating the patient on individual oral hygiene is important, this may not be sufficient [6]. It may be due to poor manual dexterity, co-morbidities, or lack of motivation [4]. In addition, gingivitis results in bleeding on brushing that further hampers oral hygiene procedures as patients try not to “traumatize” the gingiva with toothbrushing [7].

The additional use of chemicals for plaque control may facilitate the removal of and prevent microbial plaque accumulation, potentially reducing reliance on mechanical oral care methods [6]. However, the use of antiseptics is associated with a wide range of side effects [4]. In particular, the long-term use of chlorhexidine-based mouthwash, which is regarded as the most potent “gold standard” antimicrobial agent, can disturb oral microbiome balance and lead to tooth staining, burning sensation, and altered taste sensitivity, contributing to decreased patient compliance [4,8].

Probiotics, which are live microorganisms that, when administered in adequate amounts, confer a health benefit on the host [9], attract the attention of dental researchers and practitioners as an adjunctive measure to reduce biofilm accumulation and gingival inflammation [10,11,12,13,14]. The mechanism of probiotics’ action is complex and includes immune response modulation [10,15,16] and the development of colonization resistance, i.e., resistance to pathogen invasion [17].

A number of studies have confirmed the positive effects of probiotics, particularly the improvement in both clinical [10,12,18,19] and laboratory [10,20,21] parameters in periodontal disease. However, the literature on the effect of probiotics on periodontal disease is contradictory [15,22,23,24,25,26,27]. This may be due to significant heterogeneity among studies, as the effect of probiotics depends on many factors such as the strain and delivery vehicle used, mode of administration, characteristics of an individual microbiome, dosage, and duration of therapy [10,28].

*S. salivarius*, among the most prevalent oral commensals, is believed to have a major role in decreasing the proliferation of pathogenic microorganisms [29,30,31,32,33]. Some *S. salivarius* strains (K12, M18) have been shown to be effective for dental purposes in a number of studies [21,33,34,35,36,37,38,39,40,41]. Despite a growing number of studies on the effect of probiotics on oral health, there is still no consensus in the literature on the optimum duration of probiotic administration [42,43,44]. It was suggested that the duration of supplementation with probiotics containing *S. salivarius* M18 for three months is sufficient to introduce microorganisms into the oral microbiome and realize positive effects [45].

Therefore, this study aimed to evaluate the effects of three months of probiotic (the *Streptococcus salivarius* M18 strain) supplementation on gingival condition (inflammation signs and bleeding) and dental plaque accumulation.

## 2. Materials and Methods

### 2.1. Study Design

Ethical approval was obtained from the local ethics committee (Protocol no. 23-22, 17 November 2022). The study protocol followed the 1964 Declaration of Helsinki and its further amendments, as well as the CONSORT 2010 statement, and was registered on clinicaltrials.gov (NCT05919134, April 2023). This was a placebo-controlled trial with double-blinding and randomization. The study was carried out at the Department of Therapeutic Dentistry, Sechenov University, Moscow, Russia, between April 2023 and October 2023.

### 2.2. Study Population

Healthy young adults diagnosed with plaque-induced generalized gingivitis attending Sechenov University’s Dental Institute were recruited for the trial. Inclusion criteria were as follows: (1) healthy patients aged 18–25 years (males and females), (2) a minimum of 20 teeth, (3) no chronic or systemic conditions, and (4) gingivitis. Exclusion criteria included the following: (1) failure to provide informed consent; (2) signs of chronic periodontitis; (3) taking drugs or nutritional supplements containing pro- or prebiotics within 1 month prior to enrollment; (4) taking antibiotics within 3 months prior to enrollment; (5) allergies to the components of the studied supplements; (6) immune disorders or taking immunosuppressants; (7) taking other immune stimulants, antibacterials, or pro- and prebiotics during the trial; (8) refusal to use a prescribed medication; and (9) reluctance to attend check-ups.

### 2.3. Sample Size Calculation

Sample size was calculated based on the primary outcome variable (GBI) of a previous study [33] and was performed for Wilcoxon’s rank-sum test in the G*Power calculator (v. 3.1.9.6) for the two independent study groups, two-arm design. Alpha-level was set as 0.05, power was 80%, and the enrollment ratio was equal between the groups. The expected means of the bleeding index were 0.74 and 0.35 in the control and probiotic groups, respectively. Thus, each of the study groups required 28 patients for a total of 56 patients. Taking into account patients’ attrition, 62 patients were recruited.

### 2.4. Allocation and Interventions

Eligible individuals were invited to join the trial. All participants provided written informed consent after a careful explanation of the study’s aim, possible consequences, and benefits of participation in the study. All patients were provided with oral hygiene instructions at baseline.

Before the study began, a numerical code was assigned to each patient. A third-party operator randomized patients to the control group (received lozenges containing placebo; 31 patients) or the M18 group (received lozenges containing a probiotic (*S. salivarius* M18 ≥ 5 × 10^8^ CFU per lozenge (Dentoblis, MEDICO DOMUS, d.d.o., Niš, Serbia)); 31 patients) according to a table of random numbers generated by a computer program. Placebo pills were similar in size, color, and taste to the probiotic pills but contained no probiotic bacteria. At baseline, each participant received a box containing lozenges. The arms were not disclosed to the subjects or the researchers until the end of the study (allocation concealment).

All patients were to take one lozenge every evening for 12 weeks. The lozenges had to be dissolved in the mouth for 2 minutes without swallowing or chewing. Google spreadsheet marks were used to monitor lozenges intake. The intervention was followed by a 4-week washout period. 

### 2.5. Examiner Calibration

To determine the reproducibility of the results, calibration of the investigator was performed. Ten patients with gingivitis unrelated to the present study were examined twice. The GBI, GI, and Turesky’s index were assessed in one segment in each patient. The Kappa coefficient was 91%.

### 2.6. Outcome Variables

Primary outcomes were changes in gingival condition (gingival index, GI; gingival bleeding index, GBI) after 1, 2, and 3 months of lozenges administration and after a one-month washout. Secondary outcomes included changes in the Quigley–Hein plaque index (modified by Turesky et al.) after 1, 2, and 3 months of lozenges administration and after a washout. The indices were evaluated as described elsewhere [10,46,47].

### 2.7. Statistical Methods

Statistical analysis was carried out in R (v. 4.2.3 (15 May 2023), R Development Core Team, Columbia university, New York, NY, USA) using “rstatix”, “doBy”, “tidyverse”, and “ellipse” packages in RStudio v. 2023.03.0+386. To present continuous data, means (Ms), standard deviations (sds), medians, and interquartile ranges (IQRs) were calculated. To present categorical variables, counts and percentages were calculated. The normality of the distribution was checked using a Shapiro–Wilk test; the distribution sphericity was checked using a modified robust Brown–Forsythe Levene-type test. Fisher’s exact test was performed to compare gender distribution between the study groups. The GI and TQHPI values between the groups and among the timepoints were compared using a repeated-measures ANOVA with a post hoc paired *t*-test or Welch *t*-test. The GBI values among the groups were compared using Wilcoxon’s rank-sum test. The GBI values among the study timepoints were compared using Friedman’s test followed by Wilcoxon’s matched-pairs signed-rank test. Effect size was estimated by calculating Hedge’s g.

## 3. Results

A total of 155 patients were screened for eligibility. Ninety-three subjects did not meet the selection criteria.

The final sample included 62 patients and was randomly split into the M18 group (8 males and 23 females) and the control group (12 males and 19 females). 

**Figure 1:**
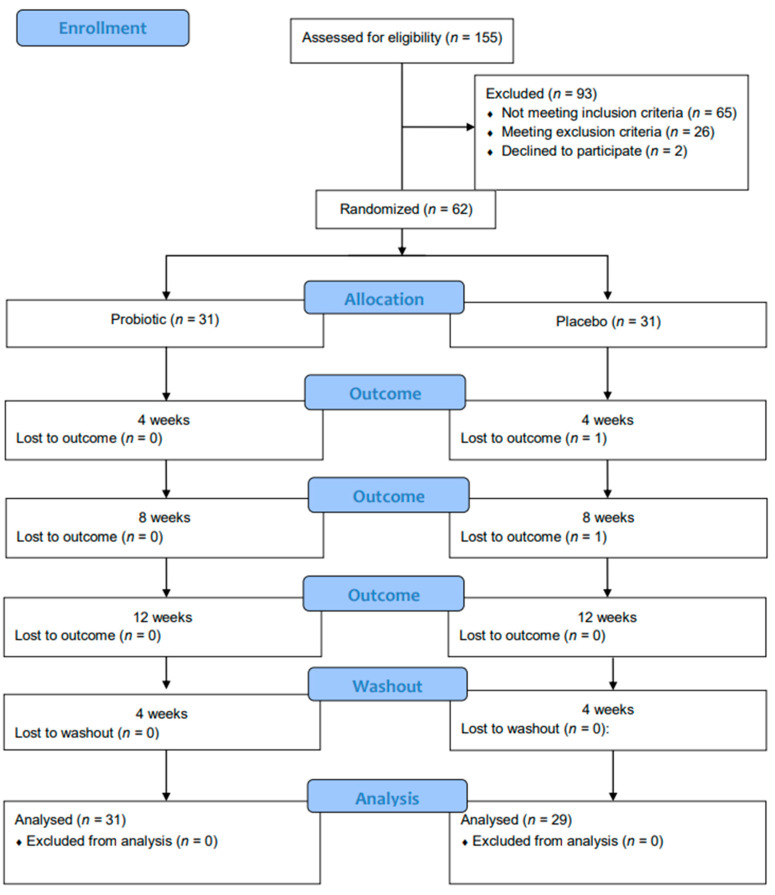
Patient flow diagram.

Two patients from the control group missed their follow-up appointments. Thus, 60 individuals eventually finished the study (31 in the M18 group and 29 in the control group) [Figure 1]. Table 1 presents information on the demographics of the study population. Group comparisons showed no differences in baseline variables.

We assessed gingival condition (GI and GBI) and biofilm accumulation (TQHPI) at baseline (T0), after one (T2), two (T3), and three months of intervention, and after the washout period, i.e., four months after the study (T4).

**Figure 2:**
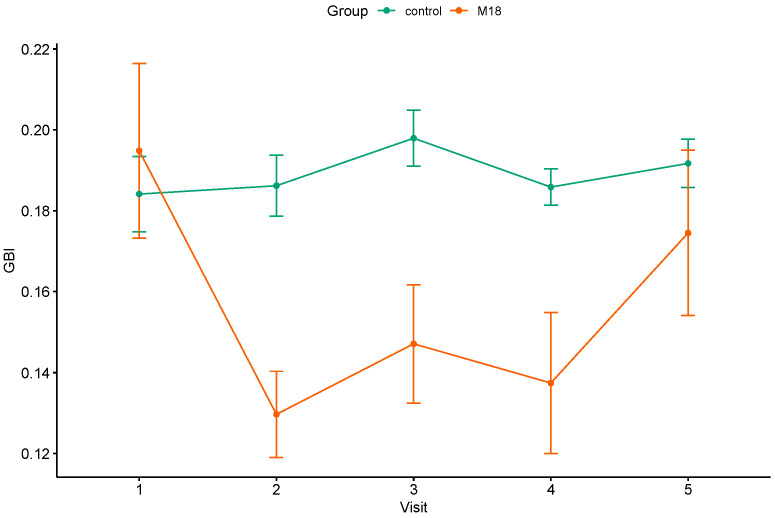
The mean trajectories of the GBI values in the study groups.

The M18 group demonstrated a significant decrease in bleeding on probing at T1, T2, and T3. The mean values of the GBI were 0.195 ± 0.12 at baseline and 0.137 ± 0.097 after 3 months of probiotic use. Participants receiving the placebo showed no difference in this parameter throughout the study (Table 2). After the washout period, the GBI scores in both groups were not significantly different from the baseline scores; however, the GBI scores at T4 were significantly higher in the control group than those in the M18 group (*p* = 0.0469). The mean trajectory of the GBI values is presented in Figure 2.

According to the ANOVA, the “visit”, the “group”, and the interaction of these factors did not have a significant influence on the GI scores (Table 3). 

The mean GI scores ranged between 0.496 and 0.562 in the M18 group and between 0.571 and 0.626 in the control group. No between-group or within-group difference was observed in the GI values (Table 4).

According to the ANOVA, the “visit” and interaction of the “visit” and “group” factors had a significant impact on the TQHPI values (Table 5).

**Figure 3:**
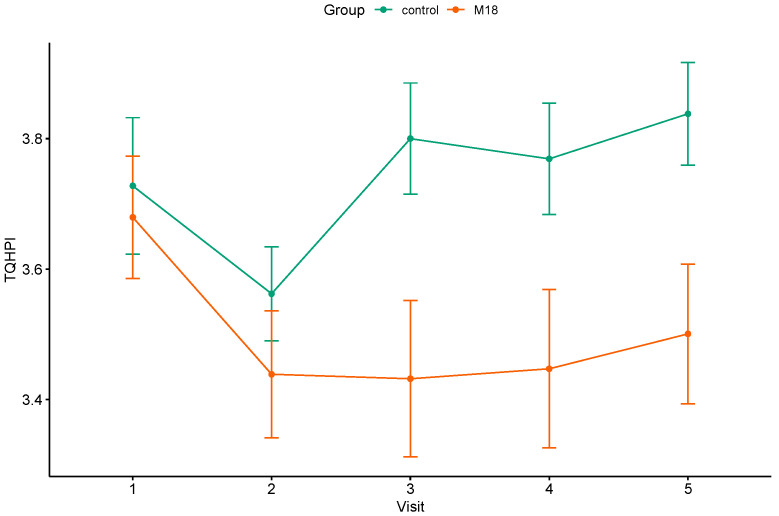
The mean trajectories of the TQHPI values in the study groups.

After 2 and 3 months of intervention, the M18 group presented a significant decrease in the TQHPI values (Table 6). However, this reduction did not sustain after the washout period (T4). The changes in the TQHPI scores in the control group throughout this study were insignificant. Effect sizes of plaque scores reduction comprised 0.63, 0.55, and 0.64 at T2, T3, and T4, respectively. The mean trajectory of the TQHPI values is presented in Figure 3.

### Adverse Events

No severe adverse effects were observed throughout the intervention. One participant in the M18 group reported subjective dry mouth; however, this side effect was temporary and did not interfere with the participant’s ability to complete the study protocol.

## 4. Discussion

We evaluated the effect of three months of probiotic (the *Streptococcus salivarius* M18 strain) supplementation on bleeding on probing, signs of gingival inflammation, and dental biofilm. Probiotic supplementation resulted in a significant decrease in bleeding on probing at 1 month, 2 months, and 3 months and a significant improvement in oral hygiene level at 2 months and 3 months. However, these changes did not sustain after the washout period.

In previous studies, *S. salivarius* M18 administration has been shown to decrease plaque index scores [34,35] and improve periodontal health indicators [33,40,41]. These effects may be explained by a number of mechanisms. An M18 *S. salivarius* strain releases a broad spectrum of bacteriocins, including salivaricins A, MPS, M, and 9 [32]. Moreover, this strain produces enzymes such as urease and dextranase, reducing the accumulation of biofilm and the pH of the environment [33]. These features of the M18 strain make it a promising candidate for prophylaxis and treatment of oral diseases.

In our previous study, the effect of a 1-month probiotic (*S. salivarius* M18) supplementation on gingival health in subjects with gingivitis was assessed. A significant reduction in the GI, GBI, and TQHPI scores was observed in the M18 group after the intervention. However, 4 weeks after the intervention, the GBI and TQHPI returned to the baseline values. It was hypothesized that a 1-month period of probiotic intake was insufficient to achieve a stable favorable effect on the oral microbiota’s composition. At the same time, Montero et al. detected a significant decrease in the levels of periodontal pathogens after 6 weeks of probiotic intake [44]. Therefore, similarly to other studies [34,45,48,49], a 3-month intervention period was chosen in the present study to achieve a long-term improvement in the gingival condition due to stable colonization.

In our study, no significant differences were detected in the GI values between the groups or among the timepoints. Similar results were reported by Benic et al., who revealed no significant influence of a 1-month probiotic *S. salivarius* M18 intervention on this parameter [18]. Burton et al. assessed the effect of the same probiotic on dental indices in children after a 1-, 3-, and 7-month supplementation [34]. Analysis of the GI values revealed no significant differences between the studied groups at all timepoints. In contrast, our previous study showed that a one-month probiotic intake resulted in a decrease in gingival inflammation according to the GI; moreover, this improvement sustained after a 1-month follow-up period [41]. Habib assessed the therapeutic potential of a complex dental probiotic containing another *S. salivarius* strain (BLIS K12). The author revealed a significant improvement in MGI scores after a four-week probiotic intake and a four-week follow-up. Surprisingly, even better results were demonstrated by the placebo group, in which the improvement in the MGI scores was registered after 2 and 4 weeks of intervention and after the follow-up period. The differences between the groups were insignificant at all timepoints [50]. 

The differences in the studies’ results and the lack of significant improvement according to the GI in many studies may be explained by the limitations of the index itself. Scoring criterion 1 is defined purely on visual assessment (a slight change in color and mild edema), which is very subjective [51]. This may particularly influence the results of the studies with the sample including patients with mild or moderate gingivitis, as the difference between scores 0 and 1 is vague [51,52]. Indeed, various studies assessing different probiotic strains reported no significant improvement in the gingival status assessed using the GI [44,53,54]. Therefore, the choice of the GI as the primary outcome to assess the effect of various treatments on the gingival condition may not be appropriate [44]. The WHO recommends using more objective criteria (such as presence of calculus, probing depth, or bleeding) in any periodontal survey [51]. Bleeding from the sulcus is known as the earliest sign of gingival inflammation [52] and is a more sensitive parameter than gingival swelling or hyperemia change. Thus, bleeding on probing is recommended to be used as the outcome in studies on gingival inflammatory disorders [51,55]. Therefore, in the present study, apart from the GI, the GBI was also used for gingival examination. Bleeding on probing significantly reduced after 1, 2, and 3 months of probiotic intake. Participants in the control group demonstrated no difference in this parameter throughout the study. On the other hand, the mean GBI scores after the washout in the M18 group were not statistically different from those at baseline. Similar results were obtained in our previous study. Participants in the M18 group exhibited a significant reduction in gingival bleeding on probing after 1 month of treatment. However, this result did not sustain after a 1-month follow-up period. These findings corroborate those reported by Scariya et al., who showed a significant reduction in the sulcular bleeding index after *S. salivarius* M18 administration for 30 days. After stopping probiotic intake, there was a significant increase in this parameter on days 45 and 60 [33].

To our knowledge, no other studies evaluated the effect of *S. salivarius* M18 on gingival bleeding on probing. In a report by He et al., this parameter was assessed after a 2-week intake of *S. salivarius* K12, and no decrease in the GI values was found [56]. A systematic review performed by Hardan et al. assessed probiotic supplementation as an adjuvant treatment in subjects with chronic periodontitis. Most of the articles included in the analysis evaluated the benefits of Lactobacillus and Bifidobacterium strains, and only one study evaluated the effect of Streptococci-containing probiotics. The latter revealed no significant differences in gingival bleeding at the study timepoints (12 and 24 weeks). However, the meta-analysis of all the studies’ results favored the intake of probiotics to reduce gingival bleeding [57].

Bleeding on probing is caused by thinning and ulceration of the sulcular epithelium, dilatation, and the increased permeability of blood vessels [58,59]. The decrease in gingival bleeding after probiotic intake may be explained by the influence on both these factors. First, *S. salivarius* strains were found to promote gingival re-epithelialization by the acceleration of the epithelial cells’ repair rate [60]. Next, these bacteria demonstrated immunomodulatory and anti-inflammatory effects [32], thus decreasing the vascular consequences of inflammation [61]. In addition, *S. salivarius* strains may provide antibiofilm activity due to the production of bacteriocins and dextranase that inhibit plaque formation [35].

In this study, a significant improvement in the oral hygiene index after 2 and 3 months of intervention in the M18-treated subjects was observed. However, these results did not sustain after the 1-month washout period. No significant changes in plaque index values were found in the control group throughout this study at all timepoints. Similarly, in our previous study, a 1-month probiotic supplementation resulted in a significant plaque reduction with further recovery to the baseline level after the washout period [41]. These findings are in agreement with those reported in some previous studies [34,35,62]. In a study by Burton et al., the M18-treated group demonstrated significantly lower mean plaque scores than those in the placebo group after a 3-month probiotic intake. The changes were more pronounced in subjects with high initial plaque scores [34]. Di Pierro et al. assessed the Cariogram outcome after 3 months of *Streptococcus salivarius* M18 supplementation in children and found a 50% reduction in the “plaque control” component of the Cariogram [35]. Similarly, Kiselnikova et al. reported a 2.2-fold decrease in plaque index scores in children after a 3-month intake of this probiotic strain [62].

On the other hand, in the studies by Benic et al. and Vesty et al., *S. salivarius* M18 intake had no [18] or minimal [63] impact on the plaque indices’ values. It may be hypothesized that it was more difficult for the probiotic strains to compete with pathogenic strains due to unfavorable changes in the oral environment associated with radiotherapy [62] or wearing orthodontic appliances [18].

We readily recognize several limitations of the current study. An issue that was not addressed was whether the intake of probiotics resulted in the colonization of the oral environment by the *S. salivarius* M18 strain, as we focused on clinical outcomes (plaque accumulation, gingival inflammation, and bleeding). Variables such as gender and diet characteristics were not factored in the study’s design. The sample mainly included patients with mild-to-moderate gingivitis, while the effect of probiotic administration could have been more prominent in subjects with severe gingival inflammation. A limited age group makes these findings less generalizable to the whole population. Further studies may focus on assessing the effects of long-term probiotic supplementation in subjects with mucositis, periodontitis, or periimplantitis. Also, further studies may be aimed at comparing the effects of probiotics as adjuncts to standard periodontal therapy as well as adjuncts to newly developed methods [64,65].

## 5. Conclusions

It may be concluded that *Streptococcus salivarius* M18 is a promising candidate strain to be used in periodontal treatment. Three-month supplementation with the probiotic significantly reduces gingival bleeding and plaque accumulation; however, a long-lasting effect is not expected, indicating the need for probiotic intake on a long-term basis.

## Figures and Tables

**Table 1:** Demographic characteristics of the study participants.

Group	M18 (*n* = 31)	Control (*n* = 29)	Statistical Significance
Sex, *n* (%)			
Female	23 (74.2)	18 (62.1)	0.4076
Male	8 (25.8)	11 (37.9)	
Age			
M (sd)	23.6 (3.8)	22.7 (4.1)	0.388
Median (Q1, Q3)	23 (22, 24)	21 (20, 24)	
Min, Max	20, 38	20, 39	

M—mean; sd—standard deviation; Q1, Q3—interquartile range.

**Table 2:** GBI values.

Timepoint	M18 (*n* = 31)	Control (*n* = 29)	Statistical Significance	Effect Size
T0				
M (sd)	0.195 (0.12)	0.184 (0.05)		
CI95%	0.151–0.239	0.165–0.203	*p* = 0.906	
Median (Q1, Q3)	0.18 (0.105, 0.300)	0.17 (0.150, 0.230)		
	a	A		
T1				
M (sd)	0.13 (0.059)	0.186 (0.041)		1.09 0.55–1.63
CI95%	0.108–0.151	0.171–0.202	*p* = 0.00004	
Median (Q1, Q3)	0.13 (0.095, 0.150)	0.19 (0.160, 0.210)		
	bc	A		
T2				
M (sd)	0.147 (0.081)	0.198 (0.037)	*p* = 0.00532	
CI95%	0.117–0.177	0.184–0.212		0.78 0.26–1.30
Median (Q1, Q3)	0.15 (0.080, 0.190)	0.21 (0.170, 0.220)		
	bc	A		
T3				
M (sd)	0.137 (0.097)	0.186 (0.024)	*p* = 0.00301	
CI95%	0.102–0.173	0.177–0.195		0.67 0.15–1.18
Median (Q1, Q3)	0.11 (0.065, 0.195)	0.19 (0.160, 0.210)		
	c	A		
T4				
M (sd)	0.175 (0.114)	0.192 (0.032)	*p* = 0.0469	0.20 −0.30–0.70
CI95%	0.133–0.216	0.180–0.204		
Median (Q1, Q3)	0.13 (0.110, 0.235)	0.19 (0.170, 0.210)		
	ab	A		

a, b, c, A: Letters show significance of differences between the timepoints in the M18 (lowercase letters) and control (uppercase letters) groups; M—mean; sd—standard deviation; Q1, Q3—interquartile range.

**Table 3:** The analysis of variance (ANOVA) table for GI scores.

Factor	DFn	DFd	F	Statistical Significance
“Group”	1.00	58.00	1.966	0.166
“Visit”	2.86	165.82	0.733	0.527
“Group”*”Visit”	2.86	165.82	1.097	0.351

DFn—number of degrees of freedom for the numerator; DFd—number of degrees of freedom for the denominator; *—interaction of the factors.

**Table 4:** GI values.

Timepoint	M18 (*n* = 31)	Control (*n* = 29)	Statistical Significance	Effect Size
T0				
M (sd)	0.562 (0.363)	0.621 (0.175)	*p* > 0.05	
CI95%	0.428–0.695	0.554–0.688		
Median (Q1, Q3)	0.46 (0.33, 0.71)	0.63 (0.50, 0.75)		
	a	A		
T1				
M (sd)	0.552 (0.200)	0.571 (0.148)	*p* > 0.05	
CI95%	0.554–0.688	0.515–0.628		
Median (Q1, Q3)	0.63 (0.42, 0.67)	0.54 (0.50, 0.63)		
	a	A		
T2				
M (sd)	0.503 (0.309)	0.595 (0.162)		
CI95%	0.389–0.616	0.533–0.657	*p* > 0.05	
Median (Q1, Q3)	0.46 (0.27, 0.67)	0.63 (0.46, 0.71)		
	a	A		
T3				
M (sd)	0.496 (0.282)	0.626 (0.160)	*p* > 0.05	
CI95%	0.393–0.600	0.565–0.687		
Median (Q1, Q3)	0.46 (0.25, 0.69)	0.71 (0.46, 0.75)		
	a	A		
T4				
Mean (sd)	0.548 (0.284)	0.611 (0.216)	*p* > 0.05	
CI95%	0.444–0.652	0.529–0.693		
Median (Q1, Q3)	0.50 (0.33, 0.71)	0.67 (0.50, 0.75)		
	a	A		

a, A: Letters show significance of differences between the timepoints in the M18 (lowercase letters) and control (uppercase letters) groups; M—mean; sd—standard deviation; Q1, Q3—interquartile range.

**Table 5:** The analysis of variance (ANOVA) table for TQHPI scores.

Factor	DFn	DFd	F-Value	Statistical Significance
“Group”	1.00	58	3.909	0.053
“Visit”	2.45	141.82	3.993	0.014 **
“Group”*”Visit”	2.45	141.82	3.470	0.025 **

** denotes significant values; DFn—number of degrees of freedom for the numerator; DFd—number of degrees of freedom for the denominator; *—interaction of the factors.

**Table 6:** TQHPI index values.

Timepoint	M18 (*n =* 31)	Control (*n =* 29)	Statistical Significance	Effect Size
T0				
M (sd)	3.679 (0.523)	3.728 (0.563)	*p =* 0.732	
CI95%	3.488–3.871	3.513–3.94		
Median (Q1, Q3)	3.65 (3.345, 4.100)	3.60 (3.300, 4.200)		
	a	AB		
T1				
M (sd)	3.439 (0.542)	3.562 (0.388)	*p =* 0.318	
CI95%	3.488–3.871	3.513–3.942		
Median (Q1, Q3)	3.50 (3.100, 3.800)	3.50 (3.300, 3.700)		
	a	A		
T2				
M (sd)	3.432 (0.667)	3.800 (0.459)	*p =* 0.0163	
CI95%	3.488–3.871	3.513–3.942		0.63 0.12–1.14
Median (Q1, Q3)	3.30 (3.025, 4.170)	3.70(3.500, 4.000)		
	b	B		
T3				
M (sd)	3.447 (0.676)	3.769 (0.459)	*p =* 0.0364	
CI95%	3.488–3.871	3.488–3.871		0.55 0.03–1.05
Median (Q1, Q3)	3.43 (2.910, 4.125)	3.70 (3.400, 4.000)		
	b	B		
T4				
M (sd)	3.501 (0.596)	3.838 (0.424)	*p =* 0.0149	0.64 0.12–1.15
CI95%	3.488–3.871	3.513–3.942		
Median (Q1, Q3)	3.43 (3.005, 4.000)	3.70 (3.600, 4.100)		
	ab	B		

a, b, A, B: Letters show significance of differences between the timepoints in the M18 (lowercase letters) and control (uppercase letters) groups; M—mean; sd—standard deviation; Q1, Q3—interquartile range.

## Data Availability

The study’s data are available from the corresponding author (K.B.) upon reasonable request.

## References

[1] Nadar B., Usha G., Lakshminarayan N. (2020). Comparative Evaluation of Efficacy of 4% Tulsi Extract (Ocimum Sanctum), Fluoridated and Placebo Dentifrices against Gingivitis and Plaque among 14–15 Years School Children in Davangere City, India—A Triple Blinded Randomized Clinical Trial. Contemp. Clin. Dent..

[2] Parkinson C.R., Milleman K.R., Milleman J.L. (2020). Gingivitis Efficacy of a 0.454% w/w Stannous Fluoride Dentifrice: A 24-Week Randomized Controlled Trial. BMC Oral Health.

[3] Sreenivasan P.K., Prasad K.V.V. (2020). Effects of a Chlorhexidine Mouthwash on Clinical Parameters of Gingivitis, Dental Plaque and Oral Polymorphonuclear Leukocytes [PMN]. Contemp. Clin. Trials Commun..

[4] Deshpande A., Deshpande N., Raol R., Patel K., Jaiswal V., Wadhwa M. (2021). Effect of Green Tea, Ginger plus Green Tea, and Chlorhexidine Mouthwash on Plaque-Induced Gingivitis: A Randomized Clinical Trial. J. Indian Soc. Periodontol..

[5] Akula S., Nagarathna J., Srinath K. (2021). Anti-Plaque and Anti-Gingivitis Efficacy of 0.25% Lemongrass Oil and 0.2% Chlorhexidine Mouthwash in Children. Front. Dent..

[6] Janakiram C., Venkitachalam R., Fontelo P., Iafolla T.J., Dye B.A. (2020). Effectiveness of Herbal Oral Care Products in Reducing Dental Plaque & Gingivitis—A Systematic Review and Meta-Analysis. BMC Complement. Med. Ther..

[7] Adam R., Ram Goyal C., Qaqish J., Grender J. (2020). Evaluation of an Oscillating-Rotating Toothbrush with Micro-Vibrations versus a Sonic Toothbrush for the Reduction of Plaque and Gingivitis: Results from a Randomized Controlled Trial. Int. Dent. J..

[8] Buakaew W., Sranujit R.P., Noysang C., Sangouam S., Suphrom N., Thongsri Y., Potup P., Usuwanthim K. (2021). Evaluation of Mouthwash Containing Citrus Hystrix DC., Moringa Oleifera Lam. and Azadirachta Indica A. Juss. Leaf Extracts on Dental Plaque and Gingivitis. Plants.

[9] World Health Organization (2006). Food and Agriculture Organization of the United Nations Probiotics in Food Health and Nutritional Properties and Guidelines for Evaluation. FAO Food Nutr. Pap..

[10] de Almeida Silva Levi Y.L., Ribeiro M.C., Silva P.H.F., Silva G.A., de Souza Salvador S.L., de Souza S.L.S., Casarin R., Júnior A.B.N., Júnior M.T., Palioto D.B. (2022). Effects of Oral Administration of Bifidobacterium *Animalis* Subsp. Lactis HN019 on the Treatment of Plaque-Induced Generalized Gingivitis. Clin. Oral Investig..

[11] Alkaya B., Laleman I., Keceli S., Ozcelik O., Cenk Haytac M., Teughels W. (2017). Clinical Effects of Probiotics Containing *Bacillus* Species on Gingivitis: A Pilot Randomized Controlled Trial. J. Periodontal Res..

[12] Krasse P., Carlsson B., Dahl C., Paulsson A., Nilsson Å., Sinkiewicz G. (2006). Decreased Gum Bleeding and Reduced Gingivitis by the Probiotic Lactobacillus Reuteri. Swed. Dent. J..

[13] Hallström H., Lindgren S., Yucel-Lindberg T., Dahlén G., Renvert S., Twetman S. (2013). Effect of Probiotic Lozenges on Inflammatory Reactions and Oral Biofilm during Experimental Gingivitis. Acta Odontol. Scand..

[14] Lee J., Kim S., Ko S., Ouwehand A., Ma D. (2015). Modulation of the Host Response by Probiotic *Lactobacillus Brevis* CD2 in Experimental Gingivitis. Oral Dis..

[15] Canut-Delgado N., Giovannoni M.L., Chimenos-Küstner E. (2021). Are Probiotics a Possible Treatment of Periodontitis? Probiotics against Periodontal Disease: A Systematic Review. Br. Dent. J..

[16] Deandra F.A., Ketherin K., Rachmasari R., Sulijaya B., Takahashi N. (2023). Probiotics and Metabolites Regulate the Oral and Gut Microbiome Composition as Host Modulation Agents in Periodontitis: A Narrative Review. Heliyon.

[17] Latif A., Shehzad A., Niazi S., Zahid A., Ashraf W., Iqbal M.W., Rehman A., Riaz T., Aadil R.M., Khan I.M. (2023). Probiotics: Mechanism of Action, Health Benefits and Their Application in Food Industries. Front. Microbiol..

[18] Benic G.Z., Farella M., Morgan X.C., Viswam J., Heng N.C., Cannon R.D., Mei L. (2019). Oral Probiotics Reduce Halitosis in Patients Wearing Orthodontic Braces: A Randomized, Triple-Blind, Placebo-Controlled Trial. J. Breath Res..

[19] Ferrer M.D., López-López A., Nicolescu T., Perez-Vilaplana S., Boix-Amorós A., Dzidic M., Garcia S., Artacho A., Llena C., Mira A. (2020). Topic Application of the Probiotic Streptococcus Dentisani Improves Clinical and Microbiological Parameters Associated with Oral Health. Front. Cell. Infect. Microbiol..

[20] Jäsberg H., Tervahartiala T., Sorsa T., Söderling E., Haukioja A. (2018). Probiotic Intervention Influences the Salivary Levels of Matrix Metalloproteinase (MMP)-9 and Tissue Inhibitor of Metalloproteinases (TIMP)-1 in Healthy Adults. Arch. Oral Biol..

[21] Moman R., O’Neill C.A., Ledder R.G., Cheesapcharoen T., McBain A.J. (2020). Mitigation of the Toxic Effects of Periodontal Pathogens by Candidate Probiotics in Oral Keratinocytes, and in an Invertebrate Model. Front. Microbiol..

[22] Gruner D., Paris S., Schwendicke F. (2016). Probiotics for Managing Caries and Periodontitis: Systematic Review and Meta-Analysis. J. Dent..

[23] Inchingolo F., Inchingolo A.M., Malcangi G., De Leonardis N., Sardano R., Pezzolla C., de Ruvo E., Di Venere D., Palermo A., Inchingolo A.D. (2023). The Benefits of Probiotics on Oral Health: Systematic Review of the Literature. Pharmaceuticals.

[24] Matsubara V.H., Bandara H.M.H.N., Ishikawa K.H., Mayer M.P.A., Samaranayake L.P. (2016). The Role of Probiotic Bacteria in Managing Periodontal Disease: A Systematic Review. Expert Rev. Anti Infect. Ther..

[25] Barboza E.P., Arriaga P.C., Luz D.P., Montez C., Vianna K.C. (2020). Systematic Review of the Effect of Probiotics on Experimental Gingivitis in Humans. Braz. Oral Res..

[26] Akram Z., Shafqat S., Aati S., Kujan O., Fawzy A. (2020). Clinical Efficacy of Probiotics in the Treatment of Gingivitis: A Systematic Review and Meta-analysis. Aust. Dent. J..

[27] Nadelman P., Magno M.B., Masterson D., da Cruz A.G., Maia L.C. (2018). Are Dairy Products Containing Probiotics Beneficial for Oral Health? A Systematic Review and Meta-Analysis. Clin. Oral Investig..

[28] Nguyen T., Brody H., Lin G.-H., Rangé H., Kuraji R., Ye C., Kamarajan P., Radaic A., Gao L., Kapila Y. (2020). Probiotics, Including Nisin-Based Probiotics, Improve Clinical and Microbial Outcomes Relevant to Oral and Systemic Diseases. Periodontol 2000.

[29] Inchingolo A.D., Malcangi G., Semjonova A., Inchingolo A.M., Patano A., Coloccia G., Ceci S., Marinelli G., Di Pede C., Ciocia A.M. (2022). Oralbiotica/Oralbiotics: The Impact of Oral Microbiota on Dental Health and Demineralization: A Systematic Review of the Literature. Children.

[30] Poorni S., Nivedhitha M., Srinivasan M., Balasubramaniam A. (2022). Effect of Probiotic Streptococcus Salivarius K12 and M18 Lozenges on the Cariogram Parameters of Patients With High Caries Risk: A Randomised Control Trial. Cureus.

[31] Hyink O., Wescombe P.A., Upton M., Ragland N., Burton J.P., Tagg J.R. (2007). Salivaricin A2 and the Novel Lantibiotic Salivaricin B Are Encoded at Adjacent Loci on a 190-Kilobase Transmissible Megaplasmid in the Oral Probiotic Strain Streptococcus Salivarius K12. Appl. Environ. Microbiol..

[32] Mato E.G., Montaño-Barrientos B.J., Rivas-Mundiña B., Aneiros I.V., López L.S., Posse J.L., Lamas L.M. (2023). Anti-caries *Streptococcus* Spp.: A Potential Preventive Tool for Special Needs Patients. Spec. Care Dent..

[33] Scariya L., Nagarathna D., Varghese M. (2015). Probiotics in Periodontal Therapy. Int. J. Pharm. Bio Sci..

[34] Burton J.P., Drummond B.K., Chilcott C.N., Tagg J.R., Thomson W.M., Hale J.D.F., Wescombe P.A. (2013). Influence of the Probiotic Streptococcus Salivarius Strain M18 on Indices of Dental Health in Children: A Randomized Double-Blind, Placebo-Controlled Trial. J. Med. Microbiol..

[35] Di Pierro F., Zanvit A., Nobili P., Risso P., Fornaini C. (2015). Cariogram Outcome after 90 Days of Oral Treatment with Streptococcus Salivarius M18 in Children at High Risk for Dental Caries: Results of a Randomized, Controlled Study. Clin. Cosmet. Investig. Dent..

[36] Park J.-A., Lee G.R., Lee J.-Y., Jin B.-H. (2023). Oral Probiotics, Streptococcus Salivarius K12 and M18, Suppress the Release of Volatile Sulfur Compounds and a Virulent Protease from Oral Bacteria: An In-Vitro Study. Oral Health Prev. Dent..

[37] Mallikarjun S.B., Chandrasekhar S.N., Salim H.P. (2021). Comparative Evaluation of Antibacterial Activity of Probiotics SK12 and SM18: An In Vitro Study. Int. J. Clin. Pediatr. Dent..

[38] Jansen P.M., Abdelbary M.M.H., Conrads G. (2021). A Concerted Probiotic Activity to Inhibit Periodontitis-Associated Bacteria. PLoS ONE.

[39] Babina K., Salikhova D., Polyakova M., Svitich O., Samoylikov R., Ahmad El-Abed S., Zaytsev A., Novozhilova N. (2022). The Effect of Oral Probiotics (Streptococcus Salivarius K12) on the Salivary Level of Secretory Immunoglobulin A, Salivation Rate, and Oral Biofilm: A Pilot Randomized Clinical Trial. Nutrients.

[40] Kiselnikova L.P., Toma E.I. (2022). Changes in the Main Dental Parameters of Preschoolers with Caries Affected by Long-Term Probiotic Intake. Pediatr. Dent. Dent. Prophyl..

[41] Babina K., Salikhova D., Doroshina V., Makeeva I., Zaytsev A., Uvarichev M., Polyakova M., Novozhilova N. (2023). Antigingivitis and Antiplaque Effects of Oral Probiotic Containing the Streptococcus Salivarius M18 Strain: A Randomized Clinical Trial. Nutrients.

[42] Horz H.-P., Meinelt A., Houben B., Conrads G. (2007). Distribution and Persistence of Probiotic *Streptococcus Salivarius* K12 in the Human Oral Cavity as Determined by Real-time Quantitative Polymerase Chain Reaction. Oral Microbiol. Immunol..

[43] Özener H.Ö., Kuru L., Kadir T., Kuru B. (2023). Bifidobacterium Animalis Subsp. Lactis as Adjunct to Non-Surgical Periodontal Treatment in Periodontitis: A Randomized Controlled Clinical Trial. Clin. Oral Investig..

[44] Montero E., Iniesta M., Rodrigo M., Marín M., Figuero E., Herrera D., Sanz M. (2017). Clinical and Microbiological Effects of the Adjunctive Use of Probiotics in the Treatment of Gingivitis: A Randomized Controlled Clinical Trial. J. Clin. Periodontol..

[45] Kaklamanos E.G., Nassar R., Kalfas S., Al Halabi M., Kowash M., Hannawi H., Hussein I., Salami A., Hassan A., Senok A.C. (2019). A Single-Centre Investigator-Blinded Randomised Parallel Group Clinical Trial to Investigate the Effect of Probiotic Strains *Streptococcus salivarius* M18 and *Lactobacillus acidophilus* on Gingival Health of Paediatric Patients Undergoing Treatment with Fixed Orthodontic Appliances: Study Protocol. BMJ Open.

[46] Wiesmüller V., Kasslatter M., Zengin B., Zotz D., Offermanns V., Steiner R., Crismani A., Kapferer-Seebacher I. (2023). Cleansing Efficacy of an Oral Irrigator with Microburst Technology in Orthodontic Patients—A Randomized-Controlled Crossover Study. Clin. Oral Investig..

[47] Kim J., Yoo S., An J., Woo J., Cho Y., Park H., Karm M. (2023). Effect of a Multichannel Oral Irrigator on Periodontal Health and the Oral Microbiome. Sci. Rep..

[48] Laleman I., Yilmaz E., Ozcelik O., Haytac C., Pauwels M., Herrero E.R., Slomka V., Quirynen M., Alkaya B., Teughels W. (2015). The Effect of a Streptococci Containing Probiotic in Periodontal Therapy: A Randomized Controlled Trial. J. Clin. Periodontol..

[49] Kraft-Bodi E., Jørgensen M.R., Keller M.K., Kragelund C., Twetman S. (2015). Effect of Probiotic Bacteria on Oral *Candida* in Frail Elderly. J. Dent. Res..

[50] Habib S. (2016). Assessment of the Therapeutic Potential of a Dental Probiotic in Orthodontic Patients Affected by Gingivitis: A Randomized Control Trial. Master’s Thesis.

[51] Benamghar L., Penaud J., Kaminsky P., Abt F., Martin J. (1982). Comparison of Gingival Index and Sulcus Bleeding Index as Indicators of Periodontal Status. Bull. World Health Organ..

[52] Ramanarayanan V., Karuveettil V., Sanjeevan V., Antony B., Varghese N., Padamadan H., Janakiram C. (2020). Measuring Dental Diseases: A Critical Review of Indices in Dental Practice and Research. Amrita J. Med..

[53] Shimauchi H., Mayanagi G., Nakaya S., Minamibuchi M., Ito Y., Yamaki K., Hirata H. (2008). Improvement of Periodontal Condition by Probiotics with *Lactobacillus Salivarius* WB21: A Randomized, Double-Blind, Placebo-Controlled Study. J. Clin. Periodontol..

[54] Iniesta M., Herrera D., Montero E., Zurbriggen M., Matos A., Marín M., Sánchez-Beltrán M., Llama-Palacio A., Sanz M. (2012). Probiotic Effects of Orally Administered *Lactobacillus Reuteri* -Containing Tablets on the Subgingival and Salivary Microbiota in Patients with Gingivitis. A Randomized Clinical Trial. J. Clin. Periodontol..

[55] Bessa Rebelo M.A., de Queiroz A.C. (2011). Gingival Indices: State of Art. Gingival Diseases—Their Aetiology, Prevention and Treatment.

[56] He L., Yang H., Chen Z., Ouyang X. (2020). The Effect of Streptococcus Salivarius K12 on Halitosis: A Double-Blind, Randomized, Placebo-Controlled Trial. Probiotics Antimicrob Proteins.

[57] Hardan L., Bourgi R., Cuevas-Suárez C., Flores-Rodríguez M., Omaña-Covarrubias A., Nicastro M., Lazarescu F., Zarow M., Monteiro P., Jakubowicz N. (2022). The Use of Probiotics as Adjuvant Therapy of Periodontal Treatment: A Systematic Review and Meta-Analysis of Clinical Trials. Pharmaceutics.

[58] Newbrun E. (1996). Indices to Measure Gingival Bleeding. J. Periodontol..

[59] Lang N.P., Joss A., Orsanic T., Gusberti F.A., Siegrist B.E. (1986). Bleeding on Probing. A Predictor for the Progression of Periodontal Disease?. J. Clin. Periodontol..

[60] Fernandez-Gutierrez M.M., Roosjen P.P.J., Ultee E., Agelink M., Vervoort J.J.M., Keijser B., Wells J.M., Kleerebezem M. (2017). Streptococcus Salivarius MS-Oral-D6 Promotes Gingival Re-Epithelialization in Vitro through a Secreted Serine Protease. Sci. Rep..

[61] Stowik T. (2016). Contribution of Probiotics Streptococcus Salivarius Strains K12 and M18 to Oral Health in Humans: A Review. Ph.D. Thesis.

[62] Kiselnikova L.P., Tsarev V.N., Toma E.I., Podporin M.S. (2021). Microbiocenosis of the oral cavity of children: Clinical and microbiological characteristics and correction with probiotics based on salivary streptococci. Clin. Dent..

[63] Vesty A., Gear K., Boutell S., Taylor M.W., Douglas R.G., Biswas K. (2020). Randomised, Double-Blind, Placebo-Controlled Trial of Oral Probiotic Streptococcus Salivarius M18 on Head and Neck Cancer Patients Post-Radiotherapy: A Pilot Study. Sci. Rep..

[64] Ramanauskaite E., Machiulskiene V., Shirakata Y., Dvyliene U.M., Irena Nedzelskiene I., Sculean A. (2023). Clinical evaluation of sodium hypochlorite/amino acids and cross-linked hyaluronic acid adjunctive to non-surgical periodontal treatment: A randomized controlled clinical trial. Clin. Oral Investig..

[65] Scribante A., Gallo S., Pascadopoli M., Frani M., Butera A. (2023). Ozonized gels vs. chlorhexidine in non-surgical periodontal treatment: A randomized clinical trial. Oral Dis..

